# Electrohydrodynamic-assisted Assembly of Hierarchically Structured, 3D Crumpled Nanostructures for Efficient Solar Conversions

**DOI:** 10.1038/srep38701

**Published:** 2016-12-07

**Authors:** Hidetaka Ishihara, Yen-Chang Chen, Nicholas De Marco, Oliver Lin, Chih-Meng Huang, Vipawee Limsakoune, Yi-Chia Chou, Yang Yang, Vincent Tung

**Affiliations:** 1 School of Engineering, University of California, Merced, California 95343, USA; 2 Molecular Foundry, Lawrence Berkeley National Lab, Berkeley, California 94720, USA; 3 Department of Electrophysics, National Chiao Tung University, Taiwan; 4 Department of Materials Science and Engineering, University of California, Los Angeles, California 90095, USA; 5 California NanoSystems Institute, California 90095, USA

## Abstract

The tantalizing prospect of harnessing the unique properties of graphene crumpled nanostructures continues to fuel tremendous interest in energy storage and harvesting applications. However, the paper ball-like, hard texture, and closed-sphere morphology of current 3D graphitic nanostructure production not only constricts the conductive pathways but also limits the accessible surface area. Here, we report new insights into electrohydrodynamically-generated droplets as colloidal nanoreactors in that the stimuli-responsive nature of reduced graphene oxide can lead to the formation of crumpled nanostructures with a combination of open structures and doubly curved, saddle-shaped edges. In particular, the crumpled nanostructures dynamically adapt to non-spherical, polyhedral shapes under continuous deposition, ultimately assembling into foam-like microstructures with a highly accessible surface area and spatially interconnected transport pathways. The implementation of such crumpled nanostructures as three-dimensional rear contacts for solar conversion applications realize benefits of a high aspect ratio, electrically addressable and energetically favorable interfaces, and substantial enhancement of both short-circuit currents and fill-factors compared to those made of planar graphene counterparts. Further, the 3D crumpled nanostructures may shed lights onto the development of effective electrocatalytic electrodes due to their open structure that simultaneously allows for efficient water flow and hydrogen escape.

The rise of graphene-based nanomaterials has fascinated scientists across many disciplines. Long-range π-conjugated networks in graphene give rise to extraordinary electrical, thermal, catalytic, and mechanical properties^1^,^2^. These properties generate huge interest in the possible implementation of graphene into a myriad of applications, including transparent conductors, electrochemical energy storage, conductive reinforced composites, sensors, and catalytic supports. Central to translating these superlative material properties into real world applications are the development of viable strategies that are capable of circumventing the layer-dependent electronic structures of graphene sheets. To this end, a number of approaches, such as template-directed synthesis, chemical vapor deposition (CVD) over a porous catalyst, and sugar blowing, have been developed to fabricate three-dimensional (3D) graphene monoliths^3^,^4^,^5^,^6^. While these synthetic approaches hold some promise, the needs for harsh processing conditions and laborious removal of the sacrificing molds inevitably introduce complexity for large-scale production and potential contamination of the functional interfaces^7^,^8^.

Alternatively, a recent transition from the consideration of graphene as a product to its treatment as a reactant offers new promise to address the aforementioned challenges^9^,^10^,^11^,^12^,^13^,^14^. One such example is the deformation of 2D graphene oxide (derived from chemical exfoliation of graphite, a.k.a., GO) into 3D crumpled nanostructures made possible by the facile aerosol assembly. In essence, rapid solvent evaporation of aerosol-generated droplets drives the tiling and clustering of GO sheets due to strong intermolecular forces, and subsequently compresses them into 3D crumpled nanostructures. Analogous to a crumpled paper ball, these crumpled nanostructures have proven compressive-resistant and can be closely packed in a bulk form without significantly compromising the intrinsic material properties, such as high free volume, accessible surface area, and specific capacity^4^,^7^,^11^,^12^,^15^. In parallel, the synergistic combination of high aspect ratio structures, favorable energetics, and mechanical and chemical robustness make crumpled nanostructures well suited for a variety of applications. These include conductive scaffolds for electrochemical capacitors^16^, adducts^17^ microbial fuel cells^11^, lubricants^17^, and hydrogen evolution reaction (HER)^18^. In this list, solar conversion applications are notably absent. Their omission owes to a combination of faults. First and foremost, the thick, graphite-like conformations emanated from the clumping of GO sheets form shunting pathways with the neighboring semiconducting active layers, thus adversely affecting the overall transport characteristics. Second, the effective contacting area of adjacent crumpled nanostructures is precipitously reduced as a result of the near-spherical geometry, serving as constricting points for conductivity. Third, the needs for multi-step transferring processes are detrimental to the electrically functional interfaces. Furthermore, post high-temperature reduction (*>*400* *°C) is required to revert the intrinsically insulating GO to conductive reduced graphene oxide (rGO). Thus, it is highly desirable to develop a facile, low-temperature, and high throughput strategy that allows for direct assembly and integration into crumpled nanostructures for micro-scale integration and macro-scale applications without forming aggregated layers/slabs and unfavorable interfaces^4^,^19^,^20^.

Recent new insights into the unique colloidal chemistry and mechanical properties of rGO open up new avenues to address the aforementioned challenges. Electrically conductive rGO can be well dispersed in aqueous solutions without the use of foreign stabilizers by manipulating the electrostatic repulsion mechanism. For example, electrostatically ionizable carboxylic edges propel rGO sheets from one another other at high pH values, thus preserving the single layer conformations in colloidal dispersions^21^,^22^. Meanwhile, previous studies, including our work on the assembly of 3D rGO networks^23^, also suggest that the atomically thin and mechanically strong π-conjugation networks can be physically deformed and rationally engineered into various shapes^3^,^24^ even through the use of a soft template such as a droplet of water^4^,^25^,^26^. Therefore, rGO can be deemed as a soft, active material that can respond to both electrostatic and mechanical stimuli. We thus surmise that if external stimuli in the form of electrostatic and capillarity-induced-mechanical cues can be rationally introduced, 3D crumpled nanostructures with electrically addressable, energetically favorable, and mechanically robust interfaces can be constructed. Here, we demonstrate that electrostatically charged droplets generated through the scalable ElectroHydroDynamic (EHD) process can be used as freestanding nanoreactors to enable the formation of 3D crumpled nanostructures. Specifically, the synergistic combination of electrostatic stabilization, and fission processes substantially suppresses the aggregation of rGO sheets, thus preserving the materials properties that only exhibit in single layer configurations. Next, rGO sheets spontaneously buckle and then self-fold into crumpled nanostructures by virtue of the loss of electrostatic stabilization. Unlike the hard and rigid crumpled balls, EHD-generated crumpled nanostructures are soft and adaptable, thus transforming into foam-like microstructures upon continuous deposition. The result is a monolith that integrates figures of merit of accessible surface area, improved electrical conductivity in both in-plane and out-of-plane directions, and most importantly enhanced transport characteristics when used as 3D back contacts for efficient solar conversions.

GO was synthesized through the modified Hummers’ method and was then reduced to rGO by reacting with hydrazine under appropriate conditions. Details about the reduction process can be found in the Method section. The rGO dispersion was further diluted to 50 μg/ml in a mixture of de-ionized water and methanol (DI-H_2_O: MeOH = 7:3, v/v). Note that the addition of MeOH helps to maintain the stable colloidal dispersion in the preparation phase. To isolate and then crumple rGO sheets, we have adapted the EHD deposition (well known for its use in electrospinning and -spraying, Fig. 1a, and Scheme S1a)^27^. For decades, EHD deposition has been mainly used to atomize liquid medium for high throughput production of thin film specimens^28^. A high electric field generated between the tip of spinneret and the conductive plate creates an EHD phenomenon that induces a tangential stress on the liquid surface, thereby deforming the meniscus into a conical shape (Taylor cone, Scheme S1b). Upon reaching the threshold electric field (0.575 kV/cm), the liquid meniscus breaks into self-dispersing, charged droplets^28^. In addition to serving as dispersing media^29^, these fine droplets can be deemed as freestanding, electrostatically charged colloidal systems that can be confirmed by two experiments that typically conducted in colloid science: high order Tyndall (HOT) and salting effects^21^. In accordance with the HOT effect, the EHD generated droplets exhibit a discernible combination of colors as a result of light scattering when illuminated with white light^30^. Also, the lyophobic rGO colloid being stabilized through electrostatic repulsion will immediately and irreversibly coagulate upon adding an electrolyte solution, such as sodium chloride (NaCl) (Figure S1). These observations collectively prove that EHD-generated droplets are indeed colloidal systems where the electrostatically charged microenvironments first and foremost facilitate stable dispersions of rGO sheets without forming aggregations. Another intriguing feature of EHD-generated droplets is the fission process. Upon evaporation-induced volumetric shrinkage, the charge density residing on the droplet surface drastically increases, ultimately exceeding the Rayleigh limit^31^. At this point, droplets will further rupture into numerous smaller ones^31^. This characteristic feature distinguishes the EHD process from aerosol assembly where evaporation of droplets directly concentrates, and subsequently clumps the rGO sheets together. Instead, each EHD-generated droplet will contain only fractional amount of rGO sheets before crumpling takes place. This also in turn reduces the possibility of forming multi-layered, graphite like configurations as schematically illustrated in Fig. 1b.

Indeed, scanning electron microscopy (SEM) images taken from the droplets near the tip reveal spatially distributed and largely separated rGO sheets (0.575 kV/cm, and deposition temperature of 45 °C), underscoring the impact of electrostatic stabilization. Atomic force microscopy (AFM) images along with a 3D profile scanned across the rGO sheets further provide a step height of ~1 nm, characteristic of single layered rGO (Figure S2). Next, instead of clustering and tiling, rGO sheets were found to separate further from each other, emanating from the fission process (0.575 kV/cm, and deposition temperature of 63 °C). Planar rGO sheets began to develop wrinkles on the basal plane and folded edges, and ultimately transformed into crumpled nanostructures when the surrounding environment reached a threshold temperature of 155 °C. In contrast to crumpled balls prepared by the aerosol approach that generates closed spheres and “hard” textures, the EHD-generated crumpled nanostructures exhibit a combination of an open structure and ripple-shaped thin walls (Scheme S1c). Transmission electron microscopy (TEM) images further show a high degree of crumpling features, including propagating ridges, sharp vertices, facets with radii of curvature of nanometer scales, which can be attributed to the defect-rich basal plane intrinsic to the chemical exfoliated adding Fig. 1c for corresponding TEM images. It is known that these spatially distributed defect sites can be viewed as pressure concentration points that initiate the propagation of networks of ridges when subjected to capillarity-induced compressive forces. However, the disparity between threshold temperatures for crumpling (155 °C for rGO and 400 °C for GO) suggests that the underlying mechanism may be associated with the distinctively different surface activities of rGO and GO colloids.

When rGO dispersions were directly deposited in the absence of the external electric field, rGO sheets initially formed the archetypal coffee-ring pattern and quickly precipitated into agglomerates even before the complete evaporation of solvent (Figure S3). This deviates from the frequently proposed mechanism, e.g., evaporation driven compressive forces progressively drive the dimensional transition of 2D sheets into 3D crumpled nanostructures^9^,^11^,^16^,^32^. In the case of aerosol-assembled crumpled nanostructures, GO retains stable dispersions within water droplets unless the coating layer of water is completely removed because of the high surface free energy (~62.1 mJ/m^2^) and negative Zeta-potential (ζ) across a wide range of pH values^33^. In other words, the formation of crumpled nanostructures predominately hinges on the rate of desiccation^9^. On the contrary, a greater number of oxygen functional groups are reduced in the case of rGO (surface energy of ~46.7 mJ/m^2^, Figure S4), and the tuning of surface charges through volatile ammonia therefore becomes responsible for stable colloidal dispersions. Upon annealing, the evaporation of ammonia gives rise to the loss of electrostatic stabilization and rGO sheets pucker to minimize the surface tension. In particular, the advent of locally folded, π-π stacked ridges serves as a structural support to stabilize the overall strained structure and suppress further crumpling as the drying process progresses^34^,^35^,^36^. Predictions from molecular dynamics (MD) simulations of crumpled rGO sheets in an aqueous medium mesh well with experimental observations are suggested in Figure S5. The potential energy first reduces drastically when self-folding emerges and then reaches a plateau when the establishment of localized ridges prevents further crumpling. In this light, the crumpling process is similar in that both GO and rGO use water as a dispersing media, but is quite independent due to the different driving forces.

The colloidal nature of EHD-generated droplets also provides facile control knobs, such as pH and associated ζ potential, for systematically tuning the morphology of crumpled nanostructures^21^,^22^. Figure 2a summarizes the ζ potential of rGO dispersions as a function of progressively increasing pH. When the pH value is under 7, rGO sheets are less charged and tend to agglomerate due to the lack of electrostatic stabilization. Nevertheless, the fission process enables the breakup of rGO aggregates, ultimately forming porous networks (Fig. 2b, pH = 3). Such highly porous, yet interconnected networks may have great uses for conductive scaffold applications^20^. When volatile ammonia is slowly added into the rGO dispersion, ζ monotonically decreases and finally reaches its zenith of −47 mV at pH 11. It is known that a surface charge below −30 mV (pH > 7) is considered a prerequisite for sufficient mutual repulsion of rGOs, ensuring the stability of colloidal dispersions. Indeed, porous rGO networks begin to crumble at pH 7 and further disintegrate into discrete, open structures at pH 11 (Fig. 2b, pH = 7 and 11). The pH-dependent disintegration of porous networks also concurrently reduced the dimensions of crumpled nanostructures as summarized in Fig. 2c. Meanwhile, the dimensions of crumpled nanostructures and the degree of crumpling as indicated by the density of ridges and vertices remained largely the same regardless of the increased concentrations of rGO in EHD-generated droplets. This can be attributed to the synergistic effects of electrostatic charges and droplet fission that effectively suppresses the formation of overlapping sheets before dimensional transition takes place, therefore reducing the possibility of forming thick rGO stacks that are known to be stiffer and harder to crumple. Even with a high initial rGO concentration (~1 mg/ml), the average size and degree of crumpling of individual crumpled nanostructures (Fig. 3a–c), and their assembly into monoliths (Fig. 3a–c) remained essentially unchanged.

In parallel, cross-sectional SEM and TEM images collectively reveal the formation of hierarchically porous yet spatially interconnected foam-like monoliths, comprised of extremely thin graphitic membranes connected by corrugated or folded walls at the edges (Fig. 3d). Specifically, these strut-like joints not only function as stabilizers to preserve the structural integrity but also serve as electrical conduits for effective electron transport across foam-like morphology^37^,^38^. Indeed, conductivity measurement confirms the establishment of electrically addressable pathways in both in-plane and out-of-plane directions. Figure S6a to S6c feature a set of optical and SEM images of our design with electrode separation channel lengths of 200 μm. The lateral conductivity of foam-like monoliths is found to be ~4.13 × 10^3^ S/m and is similar to that of laser-scribed or liquid-mediated rGO papers^39^. Meanwhile, out-of-plane conductivity measured via sandwiching monoliths of crumpled nanostructures between lithographically patterned electrodes also gives rise to a comparable value of 1.92 × 10^3^ S/m. The comparable conductivity in both in-plane, and out-of-plane directions confirms the elimination of conductivity constriction points commonly encountered in crumpled nanostructures (Figure S6d). Concurrently, monoliths of crumpled nanostructures are found to exhibit a combination of high surface area (875 m^2^/g) measured by the Brunauer-Emmett-Teller (BET) approach, and diverse porosities as shown in Barret-Joyner-Halenda (BJH) calculation (Figure S7) albeit using a low temperature and scalable processing route^40^. The prospect of harvesting these compelling material properties makes crumpled nanostructure based monoliths well suited for an active component for a wide range of practical applications, especially those pertinent to energy storage. In addition to fabricating large areas of monoliths, rectangular arrays of crumpled nanostructures can be selectively registered in a way similar to mask-assisted photolithography. Upon deposition, the motion of the charged droplets can be preferentially guided (including deflection or focusing) by a directional electric field, enabling the simultaneous transformation and selective patterning of crumpled nanostructures. Figure S8a features arrays of well-defined, rectangular patterns of SU-8 molds used to fabricate poly(dimethylsiloxane) (PDMS) stencils. Upon deposition, charged crumpled nanostructures preferentially deposited on areas that are not in contact with the PDMS stencils as shown in optical and SEM images (Figure S8b,c). The fidelity and variety of the patterns can be further improved when combining with programmable translational stages^41^,^42^. In addition, the crumpled nanostructure can withstand a combination of high temperature annealing up to ~ 450 °C in ambient conditions and even common transferring process. It is noted that, however, the extremely thin crumpled nanostructures underwent an irreversible unfolding process during rehydration.

The tantalizing utility of the crumpled nanostructures is demonstrated in their successful integration as 3D back contacts to improve the overall collection efficiency of photoanodes in photoelectrochemical (PEC) devices. In principle, the efficiency of thin film PEC cells, especially for the material systems that exhibit low minority carrier lifetimes, such as semiconducting titania oxides (TiO_2_) nanoparticles, is commensurate with the light absorption and carrier collection efficiency^43^. In this regard, exploring scalable nano-texturing processes with tunable, and high aspect ratio features is required. Thus far, carbon fiber electrodes (CFEs) represented the commonly used substrates because of their commercial availability, chemical and mechanical robustness. While the thread like morphology of each fiber is 3D on the nanoscale, the overall PEC performance is still limited by the relatively flat, microscopic 2D mesh configuration. As a result, photogenerated charge carriers within the semiconducting absorbers are mostly lost to recombination prior to reaching the CFEs. To surmount the morphological hurdle, nano-graphitic allotropes, including carbon nanotubes and graphene derivatives, have been extensively used as adducts in TiO_2_ nanoparticles matrices to relay the propagation of charge carriers across the entire photoanode by virtue of a fast electron-transfer mechanism^1^,^44^. Unfortunately, previous works have shown that, unless walking through a tight electrostatic assembly window (pH~5–6), nano-graphitic adducts irreversibly aggregate due to their hydrophobic nature, making further processing difficult. Specifically, the resulting phase separation precludes the formation of percolating graphitic networks. Here, we demonstrate the use of electrically conductive, energetically favorable and mechanically robust crumpled nanostructures with tunable aerial density, and feature shapes as the 3D back contacts for efficient solar conversions.

To this end, crumpled nanostructures were directly deposited onto CFEs without additional engineering steps. A combination of deposition time (~30 minutes), concentration of rGO (50 μg/mL, pH = 11), electric field (0.575 kV/cm), surrounding temperature of 155 °C and flow rate (4 μL/min) was found to deliver the most optimized coverage over the entire CFEs. Figure 4a,b together present a series of HRSEM images that demonstrate the uniformity, fidelity and reproducibility of the EHD deposition at different length scales as over 95% of the CFE (spraying area of 1 cm × 1 cm) is coated with crumpled nanostructures. The as-assembled crumpled nanostructures exhibit an average feature size of 200 nm and an average thickness in the range of 1 μm (Fig. 4c,d). To complete the photoanode, the 3D CFE/crumpled nanostructure electrodes were directly immersed in TiO_2_ aqueous dispersions, followed by drying on a preheated hotplate at 100 °C. As suggested in Fig. 4e, this method enables particulate TiO_2_ nanoparticles to directly infiltrate within the porous 3D crumpled nanostructures and thus forms a highly conformal and uniform coating for efficient light absorbing materials without the need for sophisticated control over surface charge and colloidal stability. The thickness of TiO_2_ coating and the structural integrity of embedded 3D crumpled nanostructures were confirmed by utilizing HRSEM imaging. Dense layers of TiO_2_ nanoparticles with thickness of 150 to 200 nm formed around the protruding tips of crumpled nanostructures (yellow dotted squares, and inset). In parallel, the structural integrity of crumpled nanostructures was further demonstrated through the energy dispersive X-ray (EDX) mapping where relevant elemental signals (carbon in red, and titanium in blue) show the spatial distribution of crumpled nanostructures propagated throughout the TiO_2_ assembly (Figure S9).

Figure 4g illustrates the setup of PEC measurements comprised of a three-electrode configuration equipped with a CFE/crumpled nanostructures/TiO_2_ based working electrode, Pt counter electrode, and Ag/AgCl reference electrode immersed in an aqueous 1.2 mM KOH electrolyte solution in tandem with a potentiometer. An energy band diagram of such a nanostructured photoanode, determined through ultraviolet photoelectron spectroscopy (UPS, Figure S10), suggests a possible transport mechanism where photogenerated holes in TiO_2_ move to the surface to oxidize water while electrons immediately transfer to spatially distributed CFE/crumpled nanostructure back contacts. Figure 4h shows the representative current-voltage (J-V) output characteristics, including the neat TiO_2_ only (gray line), electrostatically assembled rGO:TiO_2_ (orange line) and 3D CFE/crumpled nanostructures/TiO_2_ photoanodes (blue line), respectively. The measurements were taken under AM 1.5 G solar irradiation as a standard to compare the effects of electrode structure on the photocurrent generation by the TiO_2_ absorbers. As can be seen in the J-V curves and the summarized performance parameters in Table 1, all three specimens exhibit increased current densities as oxidation of water takes place at the interfaces between photoanode and electrolyte upon illumination. The neat TiO_2_ nanoparticle electrode showed a typical photoresponse, with a short circuit current (J_sc_) of 61.25 μA/cm^2^ according to Table 1, fill factor (FF) of 57.75 % according to Table 1 and an open circuit voltage (V_oc_) of −0.88 V. In principle, the enhancement of photocurrent is expected to proportionally scale with the increasing thickness of TiO_2_ active layers due to enhanced absorption. However, because of the particulate nature of TiO_2_ absorber, the probability of a photogenerated carrier propagating to the semiconductor-electrolyte interface decreases exponentially with the diffusion length of TiO_2_ (70~100 nm), suggesting that only charge carriers generated within the diffusion range will contribute to J_sc_ with the rest mostly undergoing non-radiative dissipation when propagating through grain boundaries^45^. This intrinsic constraint adversely limits the use of thicker TiO_2_ absorbers to effectively harness the solar irradiation. As for the rGO:TiO_2_ case, the inevitable aggregations of rGO formed metal-semiconducting Schottky contacts within the TiO_2_ absorber, giving rise to a moderate increase of J_sc_ (92.13 μA/cm^2^), and FF (62.6%). Alternatively, the incorporation of 3D crumpled nanostructures simultaneously enhanced the overall output characteristics without the use of thick absorbers, displaying a significantly improved J_sc_ of 178.42 μA/cm^2^, FF of 72% and V_oc_ of −0.95 V even with the relatively thin coating of TiO_2_ (150~200 nm normal to the vertically extended walls compared to that of 1 μm in neat TiO_2_ photoanode). Upon reaching the open circuit condition, the enhancement of J_sc_ extracted from the 3D CFE/crumpled nanostructures/TiO_2_ photoanode is more than 2 times higher than that of pristine TiO_2_. In particular, the 3D CFE/crumpled nanostructures/TiO_2_ hybrid photoanode also showed a steeper and prompt increase in J_sc_ with applied voltages, suggesting electron and hole pairs induced by photon absorption split more readily compared to particulate counterparts.

Another important feature of implementing crumpled nanostructures is the improved FF that stems from the synergistic effects between improved energetics and transport at the interfaces of crumpled nanostructures/TiO_2_. In Fig. 4g, it can be seen that the work function of crumpled nanostructures (~4.5 eV) is favorably positioned between the CFE and TiO_2_ conduction bands, therefore reducing the formation of energetic barriers. On the other hand, the spatially interconnected and electrically conductive crumpled nanostructures provide improved transport pathways without the formation of detrimental Schottky contacts. Indeed, we observed a substantial decrease in the series resistance (R_s_) to only 4.2 Ω when quantifying from the reciprocal value of the linear slope of the J-V curves near an open circuit condition. We also note that the output characteristics of our 3D CFE/crumpled nanostructures/TiO_2_ photoanodes are comparable to those made of atomic layer deposition (ALD) grown TiO_2_ on Si^46^. This greatly relaxes the constraints of the complex ALD processing and allows the use of cost-effective and readily available TiO_2_ nanoparticles. The magnitude of the photocurrent generation is further explored through pulse photocurrent response as a function of time (Fig. 4i). In accordance with the J-V output characteristics, 3D CFE/crumpled nanostructures/TiO_2_ photoanodes showed greatly enhanced, prompt and reproducible photoresponses. These results support the notion that photogenerated charge carriers are able to leverage the energetically favorable and spatially propagated transport pathways within TiO_2_ absorbers, underscoring the importance of efficient transport along the high aspect ratio architecture of the crumpled nanostructures as well as the shortened diffusion length within particulate TiO_2_ electrodes^1^,^47^. The much improved transport characteristics even give rise to a comparable J_sc_ on par with those made of a 15 μm thick film of TiO_2_ nanoparticles on Ti foil under the same illumination conditions^48^.

To further conclude the structural effect of 3D CFE/crumpled nanostructures/TiO_2_ architecture, we systematically varied the duration of EHD deposition to afford 3D crumpled nanostructures with different densities. The top panel of Fig. 5a shows a series of SEM images regarding the time dependent morphological evolution of 3D crumpled nanostructures. The aerial density of crumpled nanostructures increased rapidly even with a deposition time of only 15 minutes and reached the percolation threshold at 30 minutes. Further deposition, however, resulted in the rampant overgrowth of “clusters-like” assemblies. In Fig. 5b,c, we observed a clear trend of both J-V output and pulse photocurrent enhancements after the introduction of 3D crumpled nanostructures. Starting with the the neat TiO_2_ nanoparticles (0 min of crumpled nanostructure deposition, gray line), the average J_sc_ and FF are around 61.25 μA/cm^2^ and 57.75%, respectively. Incorporation of high aspect ratio crumpled nanostructures with a significantly lower aerial density already yielded enhancements in all output characteristics. When the aerial density of crumpled nanostructures approached the threshold for formation of percolation networks, both the J_sc_ and FF continuously increased (15 minutes of deposition, orange line) and ultimately saturated (30 minutes of deposition, blue line). As schematically depicted in the bottom panel of Fig. 5a, the implementation of crumpled nanostructures helps reduce the distance that photogenerated charger carriers must travel before collection. However, prolonged deposition time (60 minutes) leads to the emergence of local aggregations that not only form unwanted shunting pathways between electrolyte and CFE but also acts as recombination centers within the semiconducting TiO_2_ absorbers. Evidentially, the measured photocurrent density drastically increased at the cost of significantly reduced V_oc_ and FF (olive line). These results collectively demonstrate that the electrode architecture indeed plays an important role of enhanced device performance from 3D semiconductor scaffolds.

In summary, we demonstrated that EHD-generated droplets can be deemed as charged colloidal nanoreactors that combine two of most compelling forces in nature, e.g., electrostatic and capillary forces. Stimuli responsive 2D rGO sheets dispersed in these nanoreactors are found to undergo stages of electrostatic stabilization, fission, and spontaneous buckling, ultimately self-folding into 3D crumpled nanostructures with porous morphology. The combination of drying pattern characterization and MD simulation corroborates the experimental observation that the predominant driving force for EHD-generated crumpled nanostructures is the loss of electrostatic stabilization. In contrast to the aerosol-assembled crumpled nanostructures that typically clump into a closed sphere with a hard texture, the morphology of resulting crumpled nanostructures bears a close resemblance to the blooming snapdragon flowers with an open structure and ripple shaped petals. Upon continuous deposition, these thin and adaptable crumpled nanostructures were found to dynamically deform into non-spherical, polyhedral shapes, ultimately creating foam-like microstructures with doubly curved, saddle shaped edges. Of particular importance is the convergence of high aspect ratio, improved conductivity in all directions, highly assessable surface area and energetically favorable interfaces makes crumpled nanostructures ideal candidates for 3D back contacts for efficient solar conversion devices. Utilizing particulate TiO_2_ nanoparticles as an exemplary absorber for PEC, a nearly 300% enhancement of measured J_sc_ and a 150% enhancement of FF were observed compared to planar substrates. We attribute the significant enhancement of overall output characteristics to a combination of reduced series resistance, and improved carrier transport dynamics. The rational design of 3D crumpled nanostructures back contacts presented here also embodies a very visible nexus to many emerging photovoltaic applications, such as perovskite photovoltaics, where the formidable challenge is the requirement of both high temperature sintering process and ultrahigh vacuum conditions for crystalline TiO_2_ layers^49^,^50^. Further, the 3D crumpled nanostructures may shed lights onto the development of effective electrocatalytic electrodes due to their open structure that simultaneously allows for efficient water flow and hydrogen escape^18^.

## Methods

### Synthesis of crumpled nanostructures from rGO dispersions

rGO dispersions were synthesized based on the published method^21^. In essence, GO colloids (0.5 mg/ml, 40 ml) made from the modified Hummers’ approach was mixed with 0.1 ml hydrazine (35 wt% in water) and 0.56 ml ammonia (28 wt% in water) to adjust pH to 11 in a flask and stir in an oil bath at 95 °C for 1 hour. To create crumpled nanostructures, rGO dispersions (50 μg/mL) were fed through a customized EHD setup (Scheme S1a). Note that pH of rGO dispersions must maintain at 11 to obtain necessary electrostatic forces for isolating individual rGO sheets. In a typical experiment, solutions were fed to the spinneret (gauge 23 TW needle) by a syringe pump. Electric fields (0.575 kV/cm) were generated through a high power supply (ES 40P-20 W/DAM, Gamma high voltage research). Computerized multi-pass deposition was achieved through the integration of a x-y translational stage (Newport, moving speed 2 mm/sec). A table of detailed operating parameters, including concentration, solution feed rate, and annealing temperature, to afford crumpled nanostructures can be found in Table S1.

### Characterization of crumpled nanostructures

The morphologies of crumpled nanostructures were examined by a field emission SEM integrated with energy dispersive X-ray spectroscopy (EDX, ULTRA-55), AFM (Multimode, DI) and optical microscope (Leica DM-2500). Zeta potential was measured with Malvern Instruments’ Zetasizer Nanosystem. The conductivity measurements were made by depositing crumpled nanostructures for 40 hours on a pre-cleaned Si substrate with a thermally grown 300 nm SiO_2_ and were analyzed with a field effect transistor (FET) configuration using a semiconductor analyzer (Keithley 2400). Electrical contacts were made by thermally evaporating a combination of gold and chromium electrodes (100 nm) under a vacuum of 5 × 10^−8^ torr. The channel length (200 μm) between two electrodes was defined by using a shadow mask. The surface area measurements were carried out at a liquid nitrogen temperature on a Tristar II series. Thickness of crumpled nanostructure films was determined through cross sectional SEM images.

### Assembly and characterization of 3D crumpled nanostructure back contacts

In a typical preparation of 3D crumpled nanostructure back contacts, CFEs were pretreated with UV/ozone for 15 min to remove any organic contamination. rGO dispersions in a mixture of DI-H_2_O and MeOH (v/v, 7:3), pH at 11, an applied electric field of 0.575 kV/cm and a concentration of 50 μg/mL, a flow rate of 4 μL/min were directly deposited onto CFEs. The total deposition time was 30 minutes and the substrate was pre-annealed at 155 °C. Finally, a 5 mg/mL suspension of TiO_2_ (Anatase, 25 nm in diameter, Sigma Aldrich) in MeOH was prepared and sonicated using a VWR tabletop sonicator for 30 minutes to ensure stable dispersions, followed by iteratively immersing CFE/crumpled nanostructures electrodes for 5 times. Pt wire and Ag/AgCl were used as counter and reference electrodes, respectively. To ensure electrical contact, the CFE/crumpled nanostructures/TiO_2_ working electrode was connected through a toothless alligator clip, which was then connected to a tandem working station comprised of a CH Instruments and a photovoltaic characterization setup (QE-5 IPCE, ENLI Tech, Taiwan). 1.2 mM KOH solution was used as the electrolyte, which was made from dissolving 61.5 mg KOH (reagent grade, Sigma-Aldrich) into 900 mL DI-H_2_O and 100 mL ethylene glycol (anhydrous, Sigma-Aldrich). Ethylene glycol was added to adjust the pH value to 8 as well as increase the electrolyte conductivity. The working electrode was illuminated by a 150 W simulated Xenon light source with an AM 1.5 global illumination filter to get an intensity of 100 mW/cm^2^. Linear sweep voltammetry sequences were performed to identify the photocurrent density as well as the open circuit voltage of the devices. In addition, photocurrent densities in response with light switch tests were measured through Bulk Electrolysis with Coulometry technique.

## Additional Information

**How to cite this article**: Ishihara, H. *et al*. Electrohydrodynamic-assisted Assembly of Hierarchically Structured, 3D Crumpled Nanostructures for Efficient Solar Conversions. *Sci. Rep.* **6**, 38701; doi: 10.1038/srep38701 (2016).

**Publisher's note:** Springer Nature remains neutral with regard to jurisdictional claims in published maps and institutional affiliations.

## Supplementary Material

Supplementary Information

## Figures and Tables

**Figure 1:**
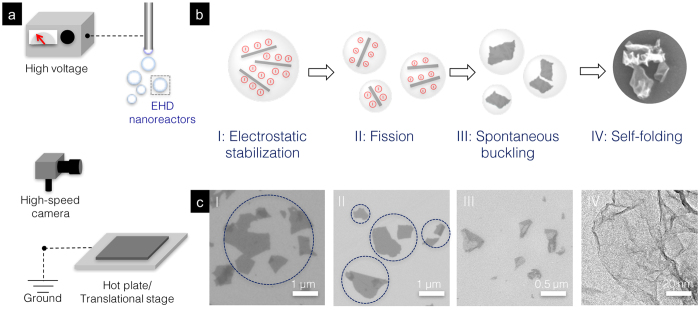
(**a**) Schematic drawing illustrates the setup of an EHD deposition for creating crumpled nanostructures. (**b**) Self-dispersing droplets can be viewed as individualized, freestanding colloidal nanoreactors that facilitate stages of (I) electrostatic stabilization of rGO, (II) fission, and (III) spontaneous buckling at both edges and basal plane or rGO, ultimately (IV) self-folding into crumpled nanostructures. (**c**) Corresponding HRSEM images corroborate the progressive transition from 2D rGO into 3D crumpled nanostructures. Specifically, HRTEM image reveals extremely thin and curved walls and a high degree of crumpling.

**Figure 2:**
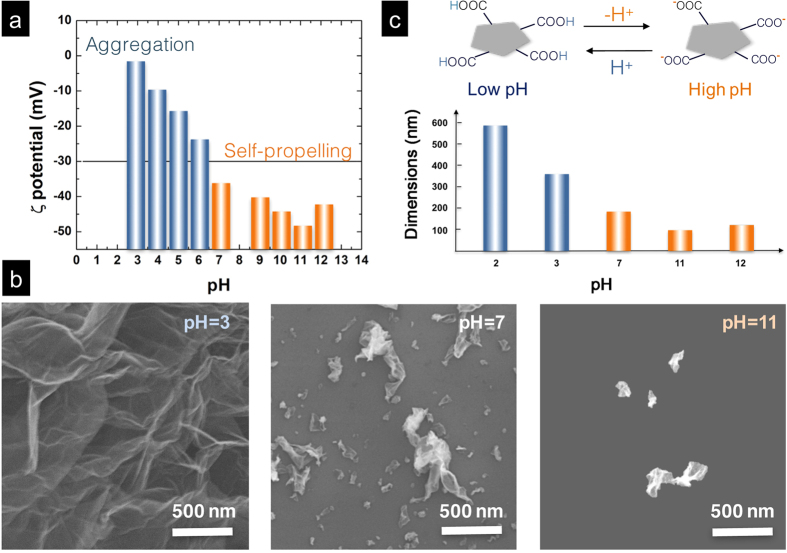
Surface properties of rGOs and their impacts on crumpled nanostructures. The colloidal nature of the charged droplets provides facile control knobs to tune the morphologies and dimensions of crumpled nanostructures. (**a**) Zeta potential of rGO colloidal dispersions as a function of pH is measured at a concentration of ~0.1 mg/ml and shows distinctively different surface properties and associated crumpling behaviors. (**b**) SEM images show pH dependent morphologies and dimensions of crumpled nanostructures. (**c**) Schematic illustration indicates the protonation and de-protonation of carboxylic groups at the edges of rGO precursors.

**Figure 3:**
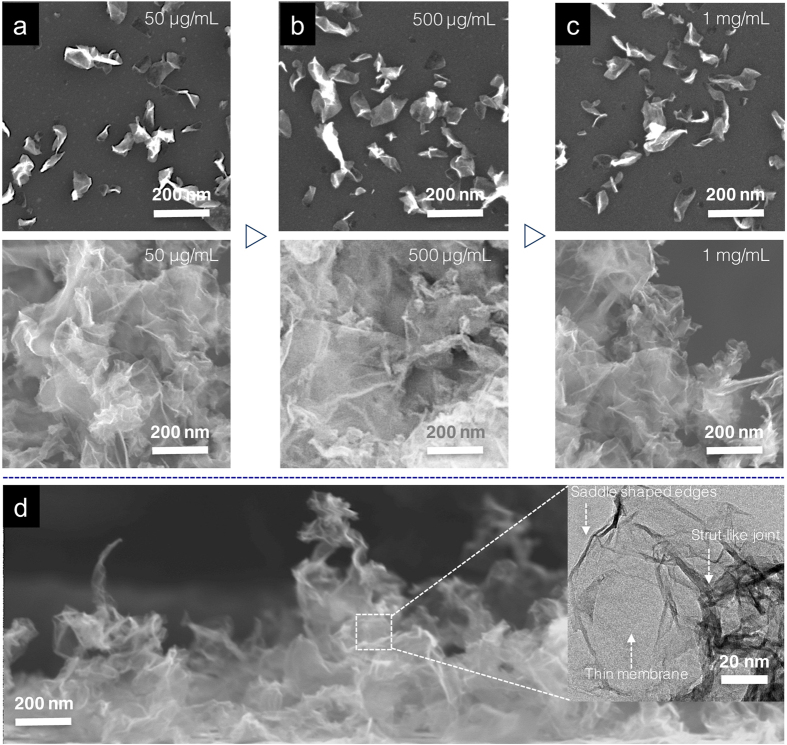
Degree of crumpling. (**a**) to (**c**) The degree of crumpling is found to be independent of the initial concentrations of rGO precursors as suggested in HRSEM images of individual crumpled nanostructures (top panel) and their assembly into foam-like monoliths (bottom panel). (**d**) Cross-sectional HRSEM image further suggests the formation of hierarchically porous and interconnected graphitic frameworks. (Inset, HRTEM image shows extremely thin and largely wrinkled walls connected by strut-like joints).

**Figure 4:**
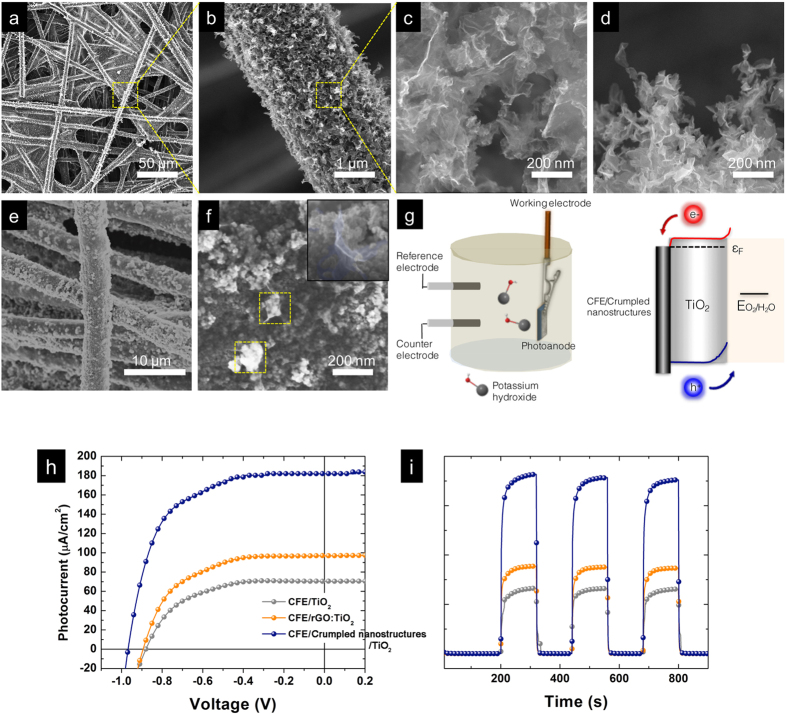
3D CFE/crumpled nanostructure back contacts for TiO_2_ based photoanodes. SEM images of crumpled nanostructures deposited on CFEs show the uniformity on (**a**) large areas, and (**b**) individual CFE. HRSEM images provide (**c**) top and (**d**) cross-sectional views of the 3D crumpled nanostructures. (**e**) The coating of TiO_2_ active layers is conformal and uniform. (**f**) Top view of the HRSEM image shows the protrusion of crumpled nanostructures as highlighted by yellow dotted rectangles. Inset displays an embedded crumpled nanostructure from a mechanically broken sample. (**g**) Representative schematic depicts the experimental setup of PEC measurements under AM 1.5G irradiation. Energy band diagram of CFE/crumpled nanostructures/TiO_2_ photoanodes suggests energetically favorable transport for electrons and holes to the opposite electrodes. (**h**) Output J-V characteristics and (**i**) time-dependent light pulse responses collectively demonstrate the much improved carrier transport at interfaces between TiO_2_ and back contacts, as is evident in the significantly improved J_sc,_ and FF.

**Figure 5:**
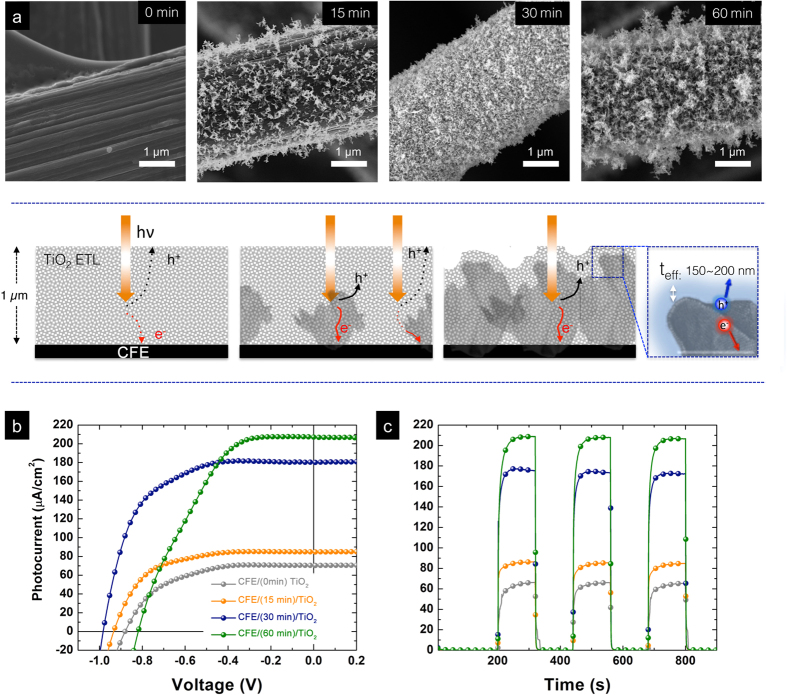
(**a**) SEM images show the progressively increased aerial densities of crumpled nanostructures as a function of deposition time (top panel). Upon continuous deposition, crumpled nanostructures gradually form percolated transport pathways (30 minutes). However, overgrowth of crumpled nanostructures (60 minutes) induces local aggregations, thus forming unwanted Schottky junctions to hamper the overall transport. (Bottom panel). (**b**) Output J-V curves and (**c**) time-dependent light pulse responses display a nearly 3x enhancement. The much improved output characteristics can be attributed to the reduced diffusion length necessary for photogenerated charger carriers before collection.

**Table 1:** J-V characteristics of TiO_2_-based photoanodes built upon 2D and 3D back contacts, respectively.

	TiO_2_	rGO: TiO_2_ (pH = 5)	Crumpled nanostructures/TiO_2_
V_oc_ [V]	−0.88	−0.89	−0.95
J_sc_ [μA/cm^2^]	61.25	92.13	178.42
FF [%]	57.75	62.60	72
R_s_ [Ω]	12.6	8.4	4.2
