# Supplementary material for: Electrohydrodynamic-assisted Assembly of Hierarchically Structured, 3D Crumpled Nanostructures for Efficient Solar Conversions
Source: Sci Rep. 2016 Dec 7;6:38701. doi: 10.1038/srep38701 (PMC5141446; doi:10.1038/srep38701)
Supplement: Supplementary Information [file srep38701-s1.pdf]

Supplementary Information:

Electrohydrodynamic-assisted Assembly of Hierarchically Structured, 3D Crumpled Nanostructures for Efficient Solar Conversions

Hidetaka Ishihara[1†], Yen-Chang Chen[1,2†], Nicholas De Marco[1], Oliver Lin[2], Chih-Meng Huang[3], Vipawee Limsakoune,[1] Yi-Chia Chou[3], Yang Yang[4,5], and Vincent Tung[1]*

[1]School of Engineering, University of California, Merced, California 95343, USA

[2]Molecular Foundry, Lawrence Berkeley National Lab, Berkeley, California 94720, USA

[3]Department of Electrophysics, National Chiao Tung University, Taiwan

[4]Department of Materials Science and Engineering, University of California, Los Angeles, California 90095, USA

[5]California NanoSystems Institute, California 90095, USA

*Email: vtung@lbl.gov

[†]These authors contributed equally to this work

Supplementary Information:

General description of the EHD deposition

Experiments were performed using a customized EHD setup. A complete diagram of the apparatus is illustrated in Scheme S1. In a typical experiment, substrates were preheated for 40 minutes prior to deposition. To avoid the formation of unwanted coffee ring effects, surface temperature was closely monitored and can be divided into various sections as highlighted in Scheme S1a. The rGO precursor solutions were fed to the spinneret by an automated syringe pump at a constant feeding rate. Upon reaching a threshold electric field, the liquid meniscus at the tip of the spinneret deformed into a conical shape. A high-speed camera was implemented to closely observe the evolution of meniscus. When a micrometric or nanometric jet disintegrated from the tip of Taylor cone to form a mist of charged droplets, a homemade shutter is removed from the substrate. The implementation of this shutter mechanism bears a close resemblance to that of thermal evaporation, preventing the deposition of unwanted impurities or large droplets in the initial stage. Deposition yield is found to be commensurate with the concentration of rGO dispersions, flow rate, and duration of EHD deposition.

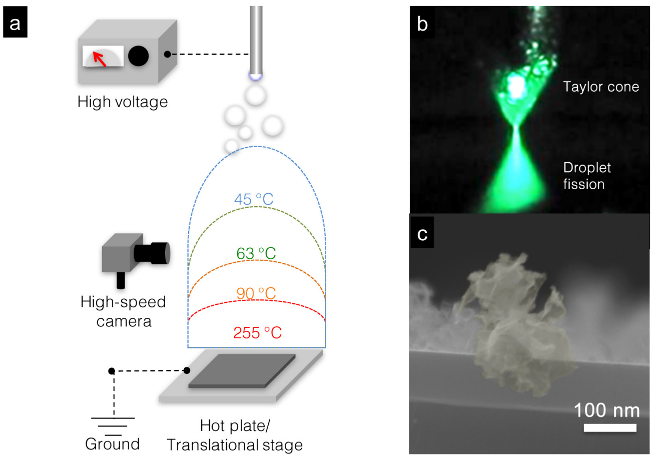

Scheme S1. (a) Schematic illustration of the EHD deposition features a programmable translational stage, a hotplate, a high-speed camera, a high voltage generator, and an automated syringe pump. (b) A representative snapshot taken from a high-speed camera captures the formation of a Taylor cone when reaching the threshold electric field (0.575 kV/cm). (c) Cross-sectional SEM image reveals compress-resistant nature of crumpled nanostructures. The structure integrity of crumpled nanostructures is preserved even upon depositing onto the hard substrate, such as silicon or glass substrates.

Supplementary Information:

Table S1 Complete parameters of transforming 2D rGO into 3D crumpled nanostructures.

Ratio [DI-H_2O: MeOH]	pH	Temperature [˚C]	Electric field [kV/cm]	Flow rate [μL/min]	Morphology
7:3	11	25	0.575	4	Flat & individual sheets
7:3	11	100	0.575	4	Wrinkles, undulations
7:3	11	125	0.575	4	Wrinkles, partially folded rGO
7:3	11	155	0.575	4	Crumpled nanostructures
7:3	11	155	0.325	4	Folded rGO with multilayered morphology
7:3	11	155	0	4	Agglomeration rGO films
7:3	7	155	0.575	4	Few layered crumpled nanostructures
7:3	2	155	0.575	4	Porous networks

Supplementary Information:

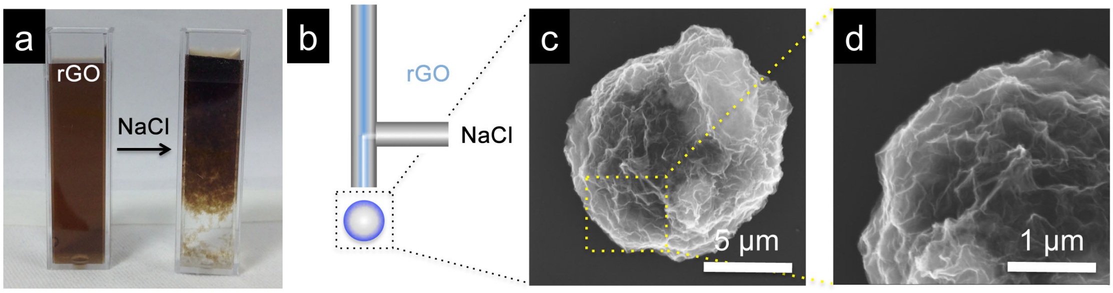

Figure S1. (a) Macroscopic salting effect induces irreversible agglomerations of rGO aqueous dispersions. (b) Schematic illustrates the setup of a coaxial EHD deposition to emulate the macroscopic salting effect at nanoscale. To this end, we have adapted the coaxial needle configuration that enables mixing of both precursor solutions, rGO in light blue and NaCl in white, just before disintegrating into fine nanodroplets. SEM images show the (c) resulting "paper ball" like morphology with (d) a thick shell and a hard texture. Adding electrolytes adversely affects the electrostatic stabilization, leading to irreversible agglomeration with highly aggregated morphology.

Supplementary Information:

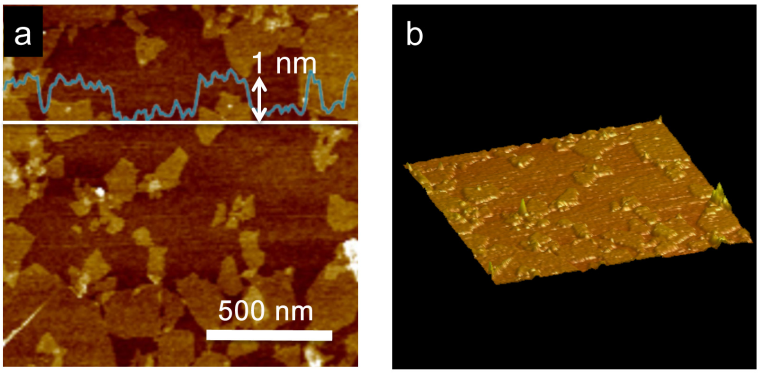

Figure S2. (a) AFM image, and (b) a corresponding 3D profile together show the spatial distribution of individual rGO sheets when the external electric field reaches 0.575 kV/cm.

Supplementary Information:

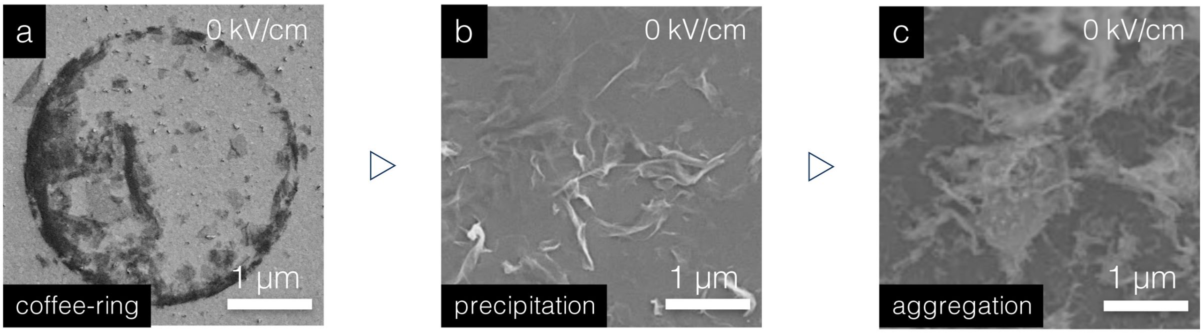

Figure S3. In the absence of an external electric field, rGO sheets initially form the coffee ring type of drying patterns, and quickly precipitate into aggregated clumps upon drying.

Supplementary Information:

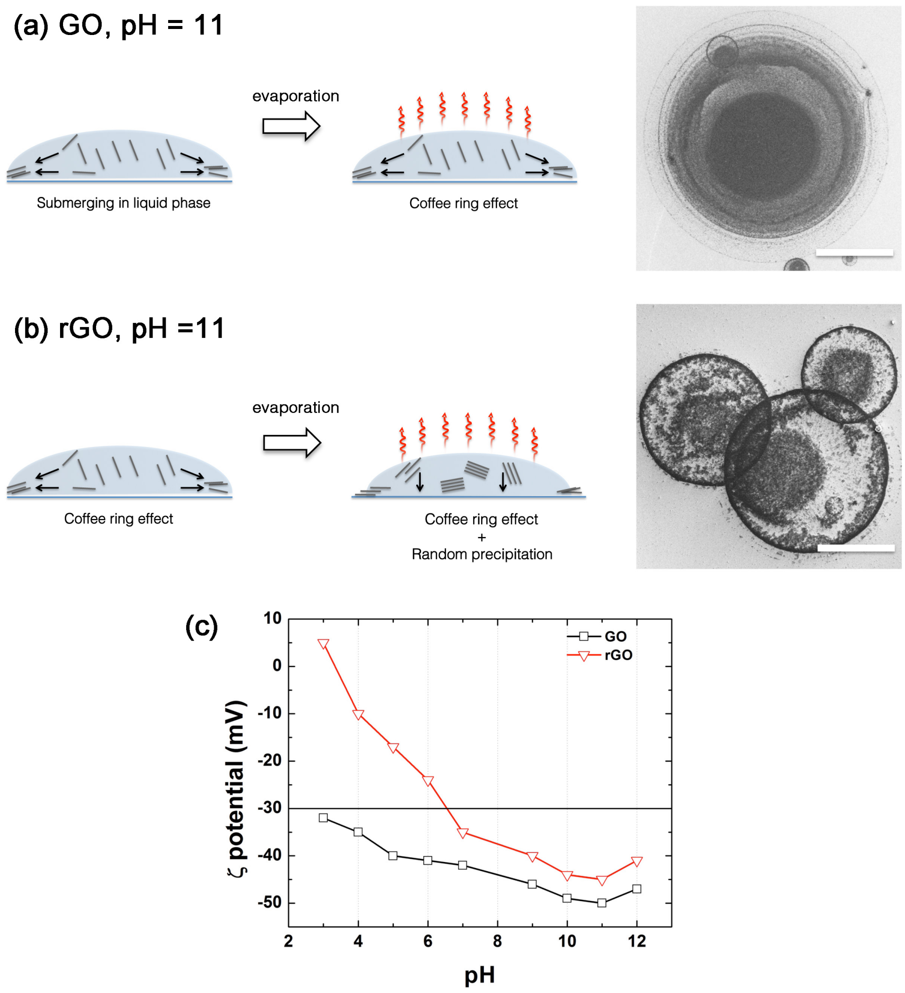

Figure S4. Surface activity of GO and rGO in aqueous dispersions at pH 11. (a) Because of the high surface energy and negative zeta potential (<-30 mV across all pH ranges), GO sheets tend to submerge within the droplets. Therefore, GO sheets leave the typical "coffee ring stain" type of drying patterns, commonly seen for aqueous colloidal dispersions. (b) In contrast, rGO sheets first develop coffee ring like drying marks as a result of negatively charged surface at pH 11. Upon evaporation, the pH value of rGO colloidal dispersions gradually reverts back to a more acidic state where surface charges drastically reduce. This leads to the irreversible and random precipitation of rGO aggregations. Scale bars are 1 μm, respectively. (c) ζ potential as a function of wide pH ranges juxtaposes the surface activity of GO and rGO sheets in colloidal dispersions.

Supplementary Information:

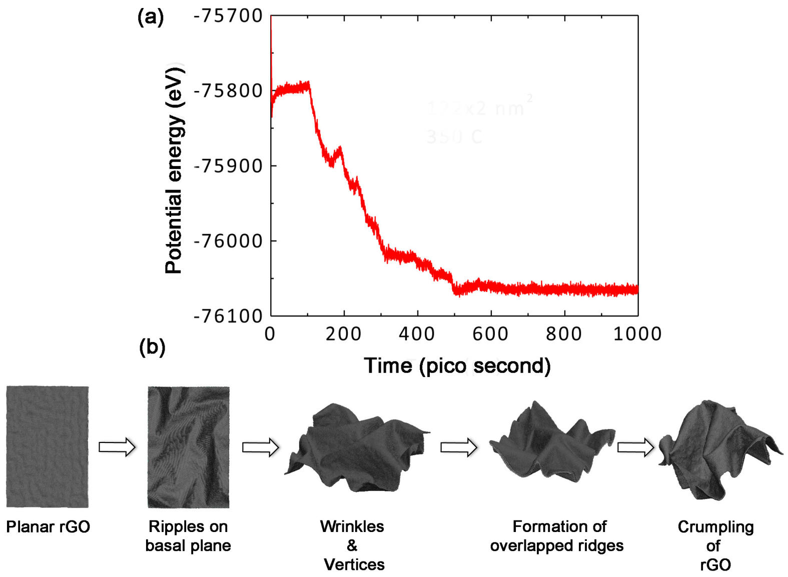

Figure S5. (a) MD simulation reveals the change of potential energy during the crumpling process of rGO. (b) Snapshots taken from MD simulation illustrate the stages of dimensional transition of a rGO sheet.

Supplementary Information:

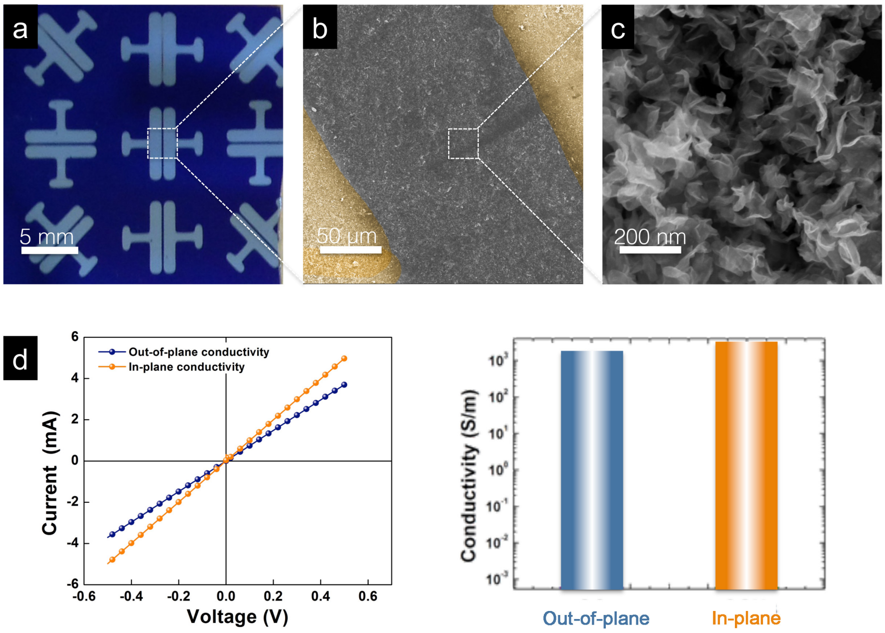

Figure S6. (a) Optical, and (b), (c) false-colored SEM images demonstrate arrays of FET electrodes used for lateral (in-plane) conductivity measurements. Densely populated crumpled nanostructures were deposited by iterative EHD depositions onto the Si/SiO_2 substrates, followed by mask-assisted thermal deposition of gold electrodes with underlying chromium adhesion layers. Spacing between two electrodes is 200 μm. (d) Output curves show the out-of-plane (blue) and in-plane (orange) conductivity of crumpled nanostructures, confirming the establishment of transport pathways in all directions.

Supplementary Information:

a b
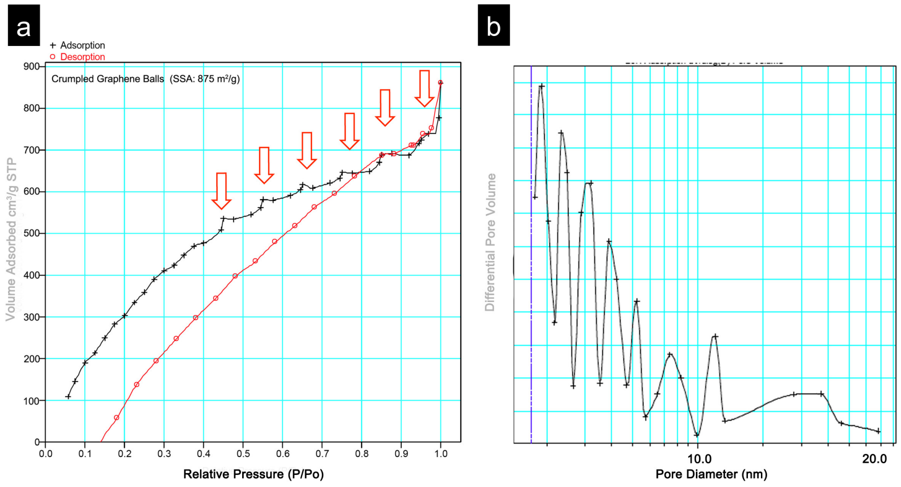

c

	EHD Synthesis	Aerosol Clumping[6]	CVD Deposition[7]	Sugar Blowing[8]
Processing temperature (˚C)	155	400	1,000	1,350
Surface area (m^2/g)	875	567	850	1,005

Figure S7. (a) BET N_2 adsorption/desorption isotherms of crumpled nanostructures, with arrows indicating various condensations along with (b) corresponding pore-size distributions based on BJH calculation. (c) Low temperature, EHD synthesized crumpled nanostructures exhibit a comparably high surface area as compared to other synthetic routes that all require high temperature annealing processes such as aerosol[6], CVD deposition[7], and sugar blowing[8], making it possible for direct integration for a myriad of applications.

Supplementary Information:

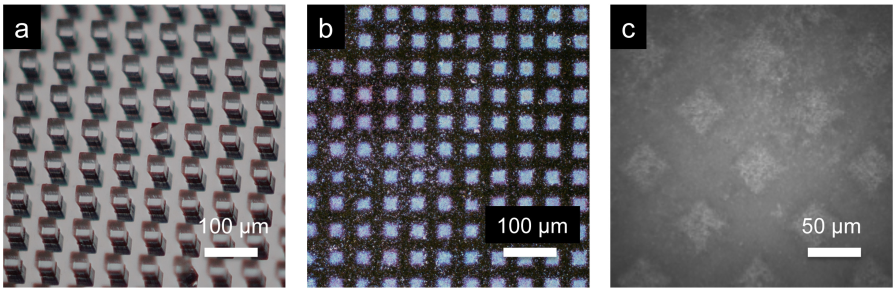

Figure S8. Selective patterning of crumpled nanostructures can be achieved through the use of a PDMS stencil. The geometry and feature size can be lithographically defined by varying the parameters of a (a) SU-8 mold. (b) Optical and (c) SEM images collectively show the selective deposition of crumpled nanostructures on the area not in a direct contact with the PDMS stencil.

Supplementary Information:

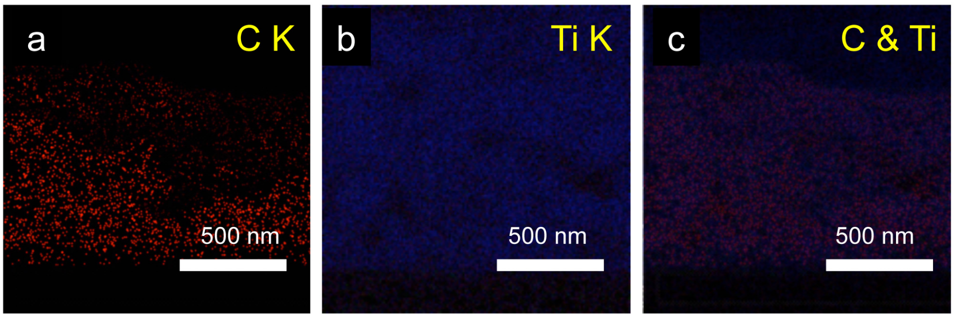

Figure S10. EDX mapping of relevant elements, (a) carbon in red, (b) titanium in blue, and (c) the combination of both, collectively suggests that the formation of percolated crumpled nanostructures, serving as scaffolds for TiO_2 growth and pathways for transport of photogenerated charged carriers.

Supplementary Information:

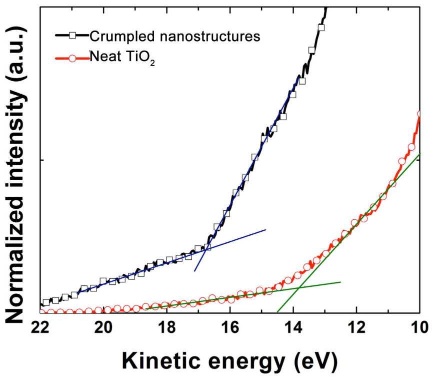

Figure S11. Energetics of crumpled nanostructures and TiO_2 nanoparticles were experimentally determined by UPS, suggesting favorable energetics of the hybrid composites.
